# Supplementary material for: PrecisionFDA Truth Challenge V2: Calling variants from short and long reads in difficult-to-map regions
Source: Cell Genom. 2022 Apr 27;2(5):100129. doi: 10.1016/j.xgen.2022.100129 (PMC9205427; doi:10.1016/j.xgen.2022.100129)
Supplement: Document S1. Figures S1–S6 [file mmc1.pdf]

Cell Genomics, Volume 2

Supplemental information

PrecisionFDA Truth Challenge V2: Calling variants from short and long reads in difficult-to-map regions

Nathan D. Olson, Justin Wagner, Jennifer McDaniel, Sarah H. Stephens, Samuel T. Westreich, Anish G. Prasanna, Elaine Johanson, Emily Boja, Ezekiel J. Maier, Omar Serang, David Jáspez, José M. Lorenzo-Salazar, Adrián Muñoz-Barrera, Luis A. Rubio-Rodríguez, Carlos Flores, Konstantinos Kyriakidis, Andigoni Malousi, Kishwar Shafin, Trevor Pesout, Miten Jain, Benedict Paten, Pi-Chuan Chang, Alexey Kolesnikov, Maria Nattestad, Gunjan Baid, Sidharth Goel, Howard Yang, Andrew Carroll, Robert Eveleigh, Mathieu Bourgey, Guillaume Bourque, Gen Li, ChouXian Ma, LinQi Tang, YuanPing Du, ShaoWei Zhang, Jordi Morata, Raúl Tonda, Genís Parra, Jean-Rémi Trotta, Christian Brueffer, Sinem Demirkaya-Budak, Duygu Kabakci-Zorlu, Deniz Turgut, Özem Kalay, Gungor Budak, Kübra Narcı, Elif Arslan, Richard Brown, Ivan J. Johnson, Alexey Dolgoborodov, Vladimir Semenyuk, Amit Jain, H. Serhat Tetikol, Varun Jain, Mike Ruehle, Bryan Lajoie, Cooper Roddey, Severine Catreux, Rami Mehio, Mian Umair Ahsan, Qian Liu, Kai Wang, Sayed Mohammad Ebrahim Sahraeian, Li Tai Fang, Marghoob Mohiyuddin, Calvin Hung, Chirag Jain, Hanying Feng, Zhipan Li, Luoqi Chen, Fritz J. Sedlazeck, and Justin M. Zook

Supplemental Figures

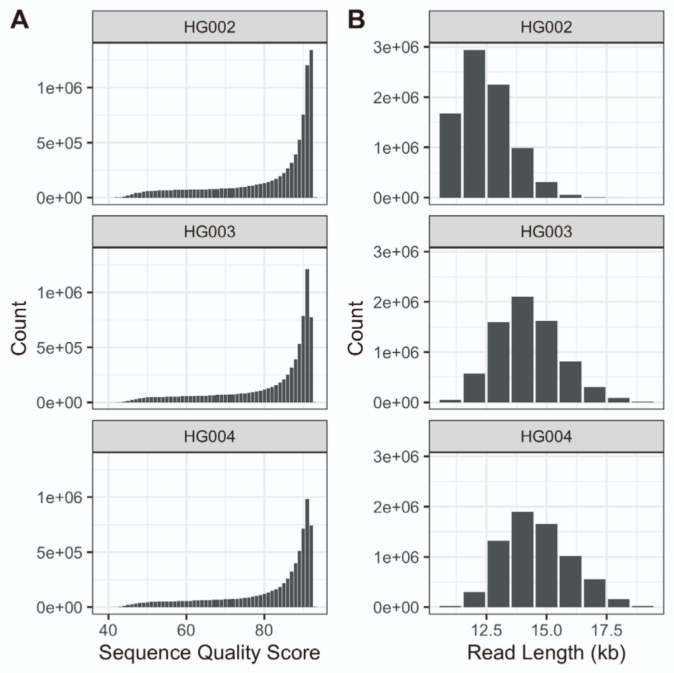

Figure S1: Read length and sequence quality score distributions for the three PacBio HiFi datasets. Sequence data metrics were calculated using FastQQ. Related to Table 1.

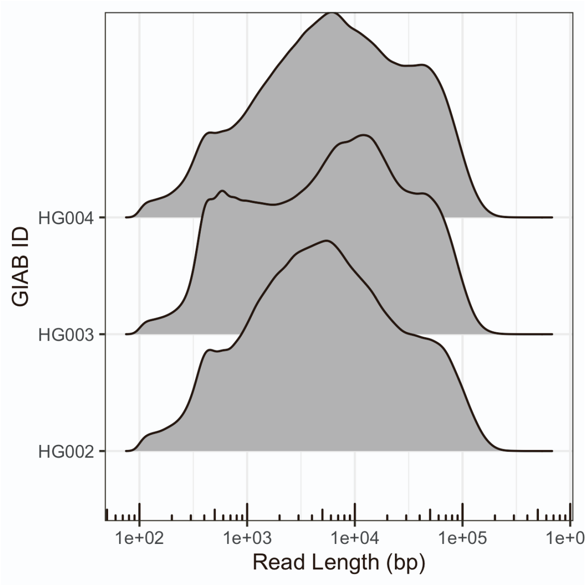

Figure S2: Read length distribution for the three ONT PromethION datasets. Related to Table 1.

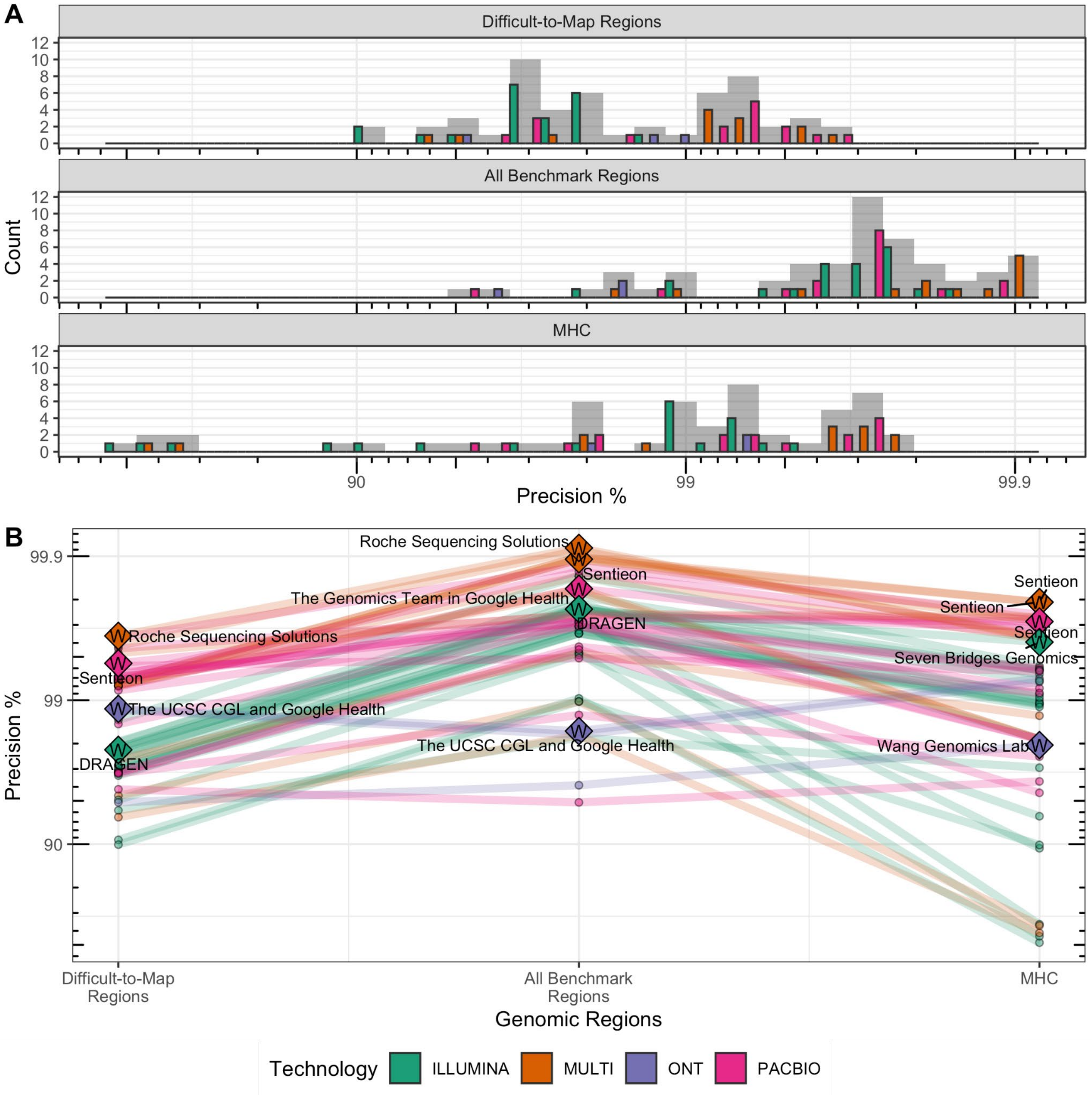

Figure S3: Precision performance (A) and submission rank (B) varied by technology and stratification(log scale). Precision is plotted on a phred scale with axes labels and ticks indicating Precision % values. Generally, submissions that used multiple technologies (MULTI) outperformed single technology submissions for all three genomic context categories. Panel A shows a Histogram of Precision % (higher is better) for the three genomic stratifications evaluated. Submission counts across technologies are indicated by light gray bars and individual technologies by colored bars. Panel B shows individual submission performance. Data points represent submission performance for the three stratifications (difficult-to-map regions, all benchmark regions, MHC), and lines connect submissions. Category top performers are indicated by diamonds with "W"s and labeled with Team names. Related to Figure 2.

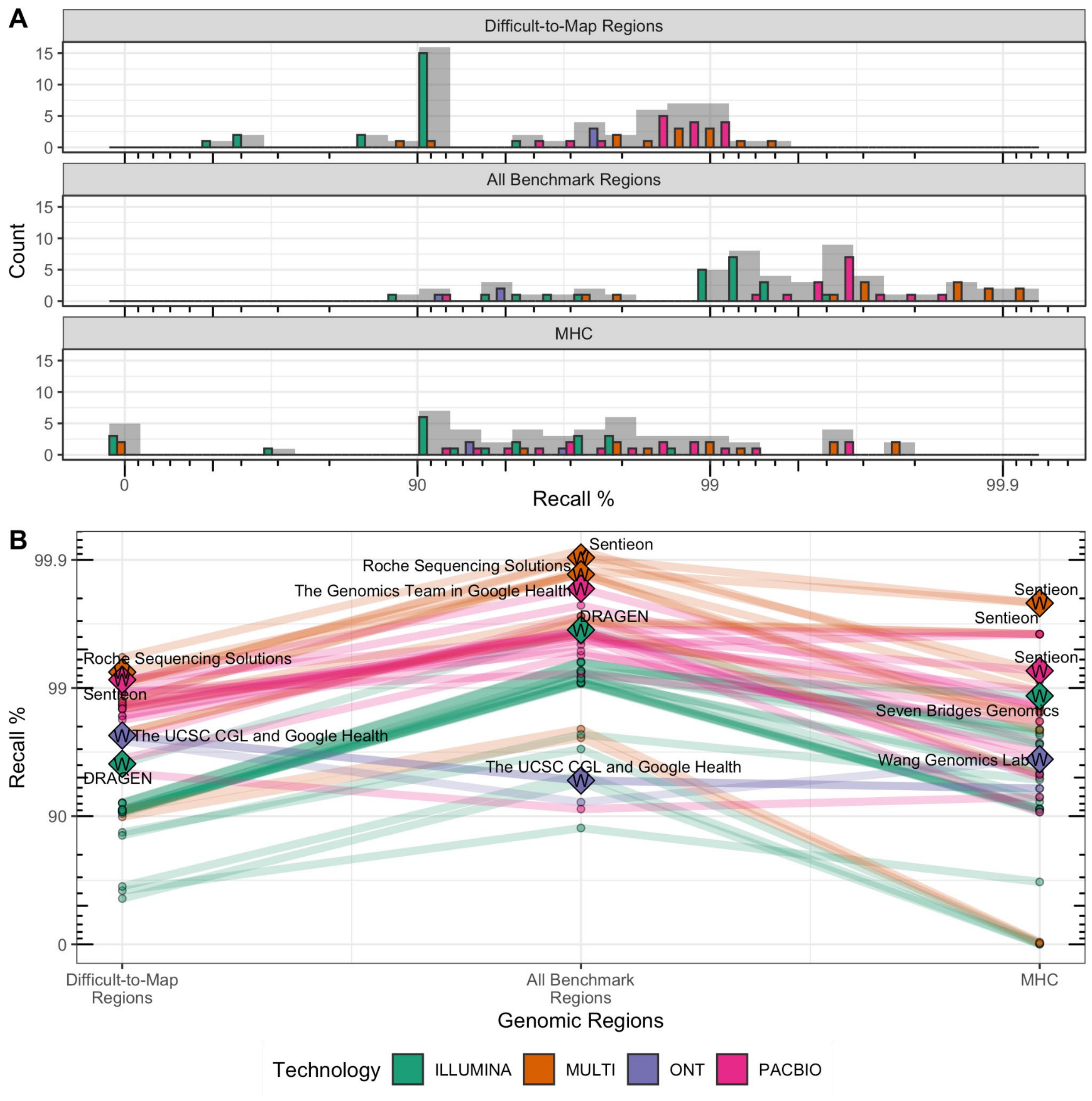

Figure S4: Recall performance (A) and submission rank (B) varied by technology and stratification(log scale). Recall is plotted on a phred scale with axes labels and ticks indicating Recall % values. Generally, submissions that used multiple technologies (MULTI) outperformed single technology submissions for all three genomic context categories. Panel A shows a Histogram of Recall % (higher is better) for the three genomic stratifications evaluated. Submission counts across technologies are indicated by light gray bars and individual technologies by colored bars. Panel B shows individual submission performance. Data points represent submission performance for the three stratifications (difficult-to-map regions, all benchmark regions, MHC), and lines connect submissions. Category top performers are indicated by diamonds with "W"s and labeled with Team names. Related to Figure 2.

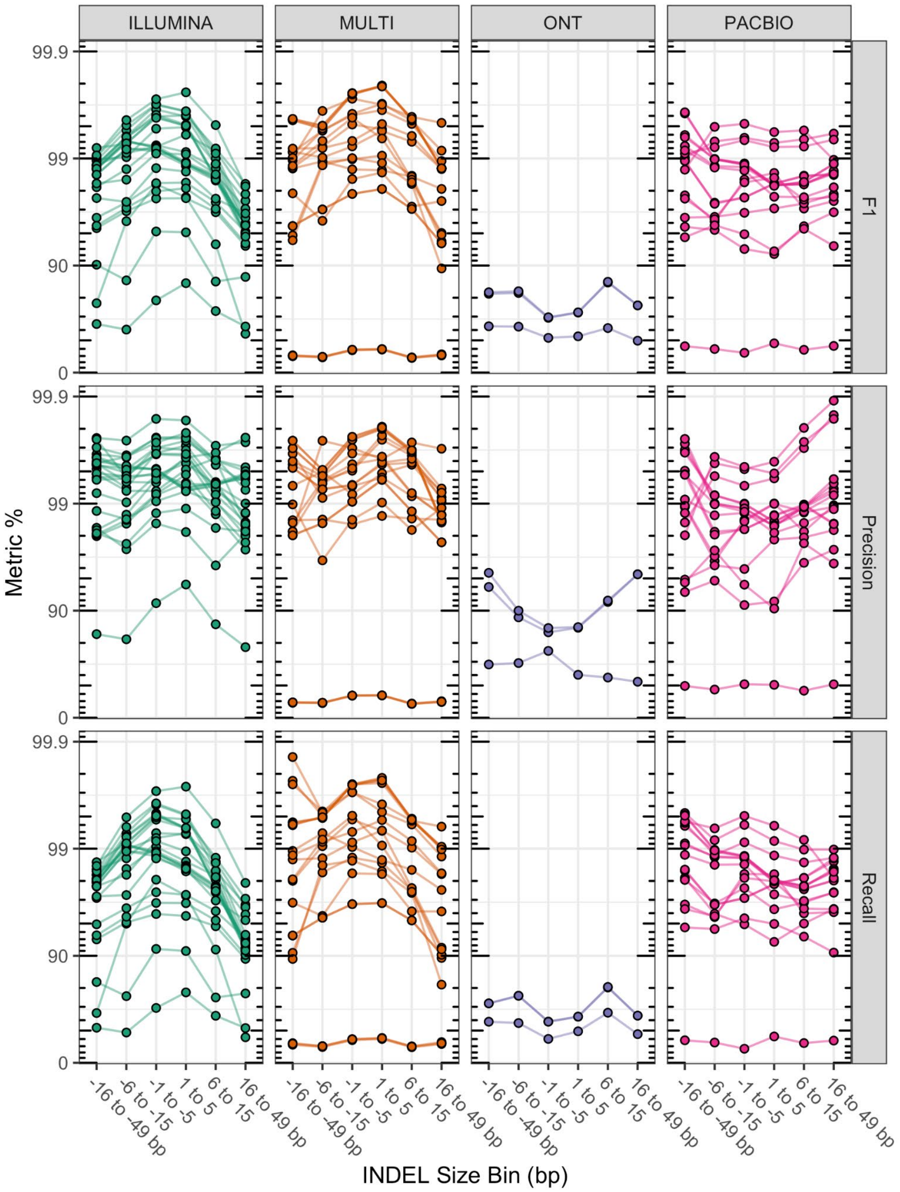

Figure S5: Variant calling performance by INDEL size varied by technology (log scale). Submission variant calling performance by INDEL size bins, with deletions indicated by negative bp size. Metric values are the geometric mean for HG003 and HG004 (the semi-blinded trio parents) with lines connecting submissions. F1 is plotted on a phred scale with axes labels and ticks indicating F1 % values. Related to Figure 2.

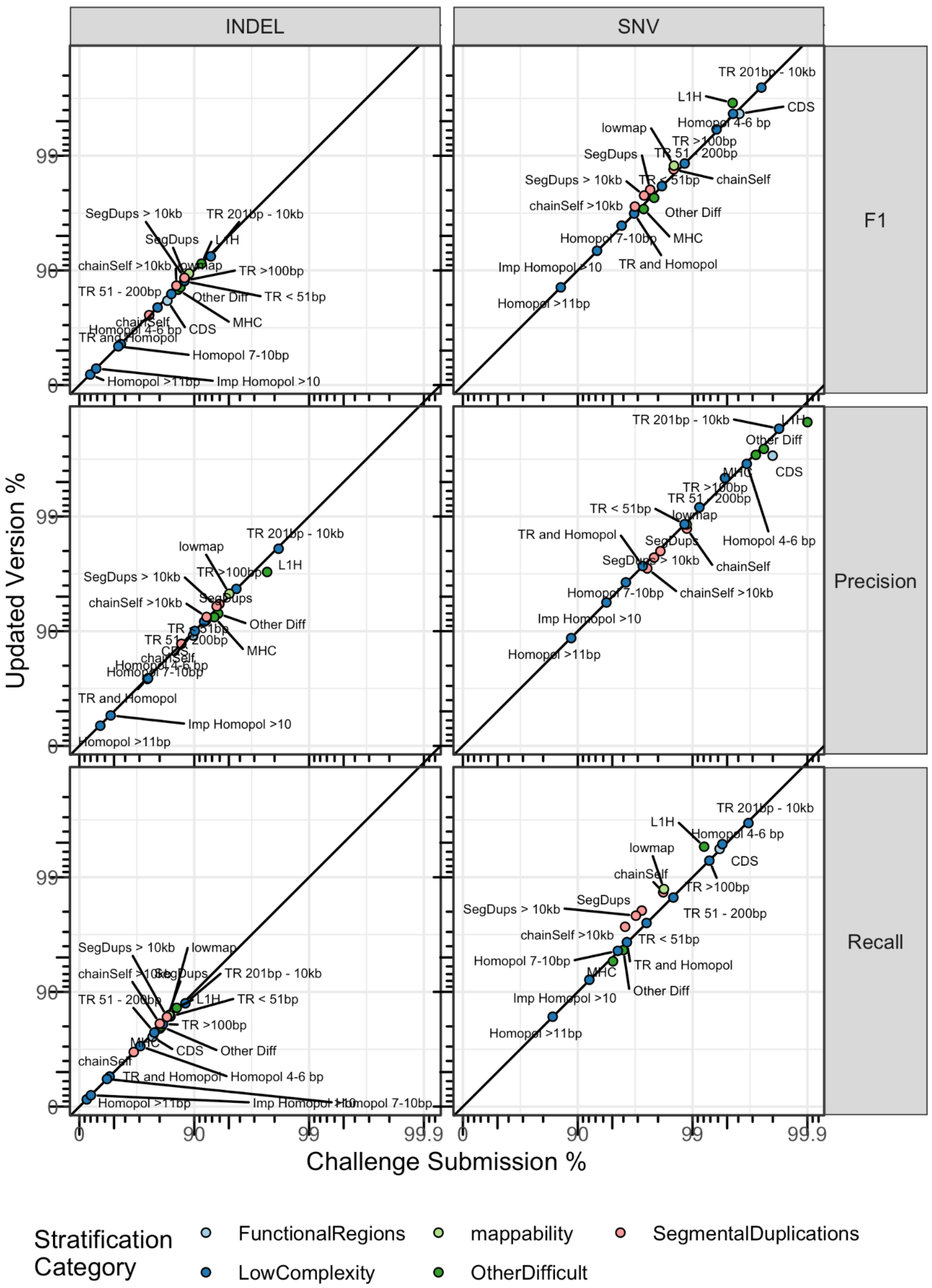

Figure S6: Comparison of submitted version of the ONT PEPPER-DeepVariant variant callset performance to an updated version. Performance metrics are plotted on a phred scale with axes labels and ticks indicating metric % values. After the challenge ended a new mapping algorithm for long read data, winnowmap, was released. Winnowmap uses weighted minimizers to improve read mapping in repetitive genomic regions (https://doi.org/10.1093/bioinformatics/btaa435). The updated variant callset utilizes this new read mapping algorithm in its pipeline. Points above and below the diagonal line indicate stratifications where the updated callset performance metric was higher than the challenge submission. The points are colored by stratification category. Related to Figure 5.
